# Validity of the Chinese multimorbidity-weighted index in measuring disease burden using health check-ups data in primary care

**DOI:** 10.1186/s12889-024-19479-6

**Published:** 2024-07-26

**Authors:** Ying-Si Lai, Xin-Yuan Gao, Wei-Hua Hu, Yi-Xuan Liu, Yong-Jin Zhang, Jia-Cong Liu, Chun Yang, Jing Liao

**Affiliations:** 1 Department of Medical Statistics, School of Public Health, Sun Yat-sen University, Guangzhou, P.R. China; 2 Sun Yat-sen Global Health Institute, Institute of State Governance, Sun Yat-sen University, Guangzhou, P.R. China; 3 Present Address: Department of Epidemiology & Biostatistics, School of Public Health, Peking University, Beijing, China; 4 Department of Chronic Disease Prevention and Treatment and Health Education, Huangpu District Center for Disease Control and Prevention, Guangzhou, P.R. China

**Keywords:** Chinese multimorbidity-weighted index, Health check-ups data, Primary care

## Abstract

**Background:**

As multimorbidity becomes common that imposes a considerable burden to patients, but the extent to which widely-used multimorbidity indexes can be applied to quantify disease burden using primary care data in China is not clear. We applied the Chinese Multimorbidity-Weighted Index (CMWI) to health check-ups data routinely collected among older adults by primary care, to examine its validity in measuring multimorbidity associated risks of disability and mortality in annual follow-ups.

**Methods:**

The study utilized data from annual health check-ups of older adults, which included information on individual age, sex, and 14 health conditions at primary care in a district of Guangzhou, Guangdong, China. The risk of CMWI for mortality was analysed in a total sample of 45,009 persons 65 years and older between 2014 and 2020 (average 2.70-year follow-up), and the risk for disability was in a subsample of 18,320 older adults free of physical impairment in 2019 and followed-up in 2020. Risk of death and disability were assessed with Cox proportional hazard regression and binary logistic regression, respectively, with both models adjusted for age and sex variables. The model fit was assessed by the Akaike information criterion (AIC), and C-statistic or the area under the receiver operating characteristic curve (AUC).

**Results:**

One unit increase in baseline-CMWI (Median= 1.70, IQR: 1.30-3.00) was associated with higher risk in subsequent disability (*OR* = 1.12, 95%*CI* = 1.05,1.20) and mortality (*OR* = 1.18, 95%*CI* = 1.14, 1.22). Participants in the top tertile of CMWI had 99% and 152% increased risks of disability and mortality than their counterparts in the bottom tertile. Model fit was satisfied with adequate AUC (0.84) or C-statistic (0.76) for both outcomes.

**Conclusions:**

CMWI, calculated based on primary care’s routine health check-ups data, provides valid estimates of disability and mortality risks in older adults. This validated tool can be used to quantity and monitor older patients’ health risks in primary care.

**Supplementary Information:**

The online version contains supplementary material available at 10.1186/s12889-024-19479-6.

## Introduction

Multimorbidity, defined as two or more chronic diseases coexisting [1], imposes a considerable burden to individuals, families and society [2]. It not only leads to poor quality of life [3], high risk of disability [4] and mortality [5], but also contributes substantially to medical costs [5, 6]. The prevalence of multimorbidity increases as people age [7]. It is estimated that over two thirds of Chinese adults aged 50 and above had multimorbidity [8].

Given over 90% Chinese older adults are cared at home and community [9], it is necessary to adopt reliable and easy-to-use tools to monitor their multimorbidity burden and to detect associated health risks at the primary care level. Primary care in China provides basic medical services, and free annual health check-ups to residents aged 65 and over in the catchment area [10]. Health check-ups include lifestyle and health status evaluation, physical examination, auxiliary examination and health education [11]. Despite rich information recorded, few studies utilized these data to estimate the prevalence of multimorbidity (i.e., ≥ 2 chronic diseases) [12], simply measured by disease count [13], overlooking the different diseases’ relative importance to health and quality of life [14]. Applying weighted multimorbidity indices that focus on specific diseases impacts provides a more distinct measure than merely recording presence or count diseases [15], enhancing the prediction of patient outcomes and the assessment of multimorbidity burden. Thus far, the extent to which widely-used multimorbidity indices can be applied to quantify primary care data is not clear. Several UK studies have used the Elixhauser comorbidity index (ECI), which includes 30 conditions associated with inpatient mortality, cost, and length of stay [16] and Charlson comorbidity index (CCI), which weights 19 conditions from a limited sample of hospitalized patients [17], to estimate mortality risks at primary care [18, 19]. Additionally, the Cambridge Multimorbidity Score [20], based on 40 long-term conditions weighted for predicting mortality, hospitalization, and primary care usage, has been used to estimate both healthcare service utilization and mortality risks [21]. However, these indexes, derived from either hospital inpatient data with mortality as the only outcome or primary care records from the Clinical Practice Research Data with healthcare service utilization and mortality risks as outcomes, turn to be less sensitive in detecting disease burden and functional decline among older community dwellers compared to general population-based indices [22, 23], like the multimorbidity-weighted index (MWI) [14] and its Chinese version CMWI [24]. The Chinese Multimorbidity-Weighted Index (CMWI) was tailored for Chinese middle-aged and older community-dwelling individuals and validated using high-quality micro household survey data [24, 25]. Evidence for the most appropriate multimorbidity indices in specific scenarios can help compare research results across different settings and outcomes [26]. However, the validity of CMWI in primary care settings has yet to be studied.

To address this gap, the aim of this study is to assess the validity of CMWI in measuring multimorbidity burden and health risks of Chinese older adults, utilizing their annual health check-ups data of primary care. Specifically, we applied the CMWI [24], developed and validated using Chinese ageing population representative cohorts [27, 28], to routinely collected health check-ups data of primary care, examining its validity in estimating multimorbidity and associated risks of disability and mortality. Establishing the validity of CMWI for primary care would facilitate its incorporation into health management of Chinese older adults.

## Methods

### Data sources and study sample

The health check-ups data of Chinese older adults from January 1, 2014 to December 31, 2020 was extracted from electronic health records of all primary care centres registered in a whole district of Guangzhou, Guangdong. This district has 5.56% older adults aged 65 years and above, covering both urban and rural residents [29]. Health check-ups include a structured questionnaire on lifestyle, health condition, self-care capacity, as well as physical examination and laboratory tests [30]. Our main study sample consisted of 45,009 older adults who participated in at least one health check-up for mortality risk analysis during the study period (01/01/2014-12/31/2020), with their first record considered as the baseline assessment. Given that disability records have only been available since 2019, we excluded 376 (2.02%) individuals with missing disability records in 2020 from those who participated in health check-ups and had no disabilities in 2019. A subsample of 18,230 older adults free of physical impairment during 01/01/2019 to 12/31/2019 with follow-up data during 01/01/2020 to 12/31/2020 was used to examine one-year disability risk. A flow chart of the selection of study population is avalable in Supplementary Fig. 1. In addition, to examine whether there were differences between the groups before and after excluding individuals with missing disability records, we conducted univariate analyses on age, sex, and CMWI for both groups, and found no statistically significant differences (Supplementary Material Table 1). Thus, exclusion of missing disability information may impact little on the corresponding results.

**Table 1:** Baseline characteristics of study sample

Characteristics	Total (*N* = 45,009)
Person-years of follow-up	121,432.70
Mean follow-up years	2.70
Age (Median, IQR)	70, 66–75
Gender , *N* (%)	
Male	19,405 (43.11)
Female	25,604 (56.89)
CMWI continuous (Median, IQR)	1.70, 1.30-3.00
CMWI category^a^,* N* (%)	
Mild	10,439 (23.19)
Moderate	28,386(63.07)
Severe	6184 (13.74)
Chronic diseases, *N* (%)	
Stroke	552 (1.23)
Memory-related disease	74 (0.16)
Cancer or malignant tumour	317 (0.70)
Asthma	28 (0.06)
Arthritis or rheumatism	2,436 (5.41)
Emotional, nervous, or psychiatric problems	196 (0.44)
Heart disease	15,844 (35.20)
Chronic lung diseases	424 (0.94)
Hypertension	26,552 (58.99)
Kidney disease	2,991 (6.65)
Diabetes or high blood sugar	9,700 (21.55)
Stomach or other digestive disease	4,607 (10.24)
Dyslipidaemia	16,895 (37.54)
Liver disease	8,036 (17.85)
Disability, *N* (%)^b^	247 (1.35)
Death, *N* (%)	1,173 (2.61)

^a^ CMWI scores of < 1.30 = mild multimorbidity burden; 1.30–3.80 = moderate multimorbidity burden; ≥3.80 = severe multimorbidity burden

^b^ For disability, participants’ CMWI in 2019 utilized as a baseline

### Multimorbidity measurement

We measured multimorbidity using the CMWI [24]. The CMWI score included 14 chronic conditions, each condition ranges from 0.20 to 5.10 points to sum an index score, reflecting their impact on physical functioning. A table detailing the CMWI disease weightings is provided in Supplementary Material Table 2. Corresponding chronic diseases were extracted from the “primary existing health problems”and “health status evaluation” parts of the health check-up records through text recognition. If a corresponding disease character was found, the condition was recorded as present; otherwise, it was coded as 0. Given the requirement of primary care providers for a simplified multimorbidity assessment in risk stratification, we classified multimorbidity burden into three categories (mild, moderate, and severe) by creating three equally spaced CMWI intervals based on the 2018 wave of the China health and retirement longitudinal study (CHARLS) dataset, which was used to develop the CMWI. In addition, we calculated the other commonly used multimorbidity indices: the multimorbidity-weighted index (MWI) [14], the Elixhauser comorbidity index (ECI) [16], the Charlson comorbidity index (CCI) [17], and Count, based on the recording of 14 disease statuses and the corresponding weights given by the indices, and categorized in a similar manner.

**Table 2:** The predictive validity of the CMWI for mortality and disability risks in 65 + older adults

	Mortality					Disability			
	*HR* (95%*CI*)	*P*		AIC	C-statistic	*OR* (95%*CI*)	*P*	AIC	AUC
CMWI, continuous	1.18 (1.14,1.22)	< 0.001		21,342	0.76	1.12 (1.05,1.20)	< 0.01	2,186	0.84
CMWI, categories				21,342	0.76			2,191	0.84
Mild [0, 1.30)	1.00	-				1.00	-		
Moderate [1.30, 3.80)	1.32 (1.12,1.55)	< 0.001				1.56 (0.94,2.79)	0.11		
Severe [3.80, 15)	2.52 (2.07,3.05)	< 0.001				1.99 (1.16,3.63)	< 0.05		

### Outcomes variables

The main outcomes were mortality and disability. All-cause mortality data for the study period (01/01/2014-12/31/2020) were obtained from the district’s Registration of Death, with a total of 7,698 records. Causes of death were recorded by using the 10th revision of the International Classification of Diseases (ICD-10). Health check-up data were linked with death records by matching unique personal identity codes. Survival time was calculated as the interval between the date of death and the date of the first health check-up during the study period. Disability was assessed as a binary variable, defined as the presence of mild and higher dependence on self-care capacity (i.e., scored > 3) [31]. Assessment of disability was based on self-care capacity assessment which is derived from the Health management technical protocol of aged issued by the National Health and Family Planning Commission of China, and consists of five activities: feeding, bathing, dressing, toileting and continence, and mobility [31]. Participants’ responses were aggregated and classified as independent (scored 0–3), mild dependence (scored 4–8), moderate dependence (scored 9–18), and unable to care for oneself (scored 19 and over) in electronic health records.

### Statistical methodology

Cox proportional hazard regression was used to estimate mortality risk of the baseline CMWI, adjusting for age and sex [32]. Continuous and categorical CMWI were used as predictors respectively. The Kaplan-Meier method was utilized to plot the overall survival curve, and the log-rank test was employed to compare the differences between categorical CMWI groups [33]. Binary logistic regression model was used to measure one-year disability risk, with either continuous or categorical CMWI, adjusting for age and sex [34]. Similar analyses were also conducted using the other four multimorbidity indexes (i.e., MWI, ECI, CCI, and Count) for both outcomes. Model fit was assessed using the Akaike Information Criterion (AIC), computed from the log likelihood and the number of model parameters, with the model having the lowest AIC selected as the best [35], and predictive capability was evaluated using the C-statistic [36] and the area under the receiver operating characteristic curve (AUC) [37]. In addition, we examined age interaction terms for CMWI regarding mortality and disability risk and found no statistically significant differences in disability risk, but a statistically significant differences in mortality risk (Supplementary Table 3). So in the sensitivity analysis, we conducted analyses of mortality risk using both continuous and categorical CMWI for participants aged 70 and older. All statistical analyses were performed using version R 4.1.3.

## Results

**Fig. 1:**
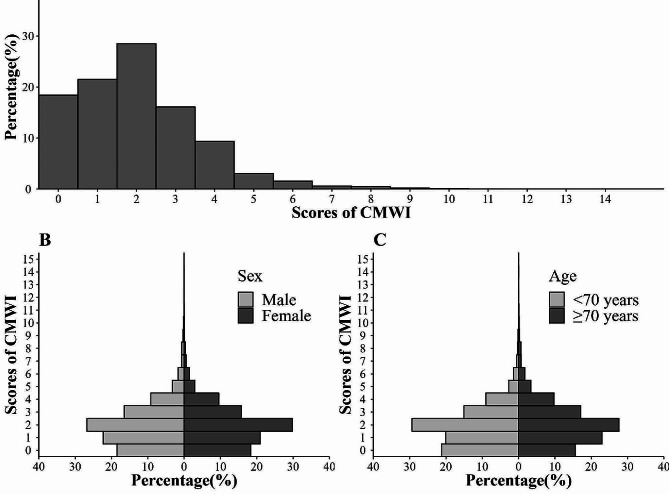
Distribution of CMWI for baseline population (**A.** Total population; **B**. Subgroup population by sex; **C.** Subgroup population by age)

In total, 45,009 participants were included in the analyses concerning mortality. Of them 1,173 (2.61%) died during the follow-up. The median age was 70 years (Inter-Quartile Range, IQR = 66–75) and 56.89% of the participants were female. The median of CMWI was 1.70 (IQR = 1.30-3.00). The total follow-up time was 121,432.70 person-years, with average 2.70 years follow-up. Among the subsample (*n* = 18,230), 247 (1.35%) developed disability in 2020 (Table 1). The distribution of the CMWI in the baseline population showed right-skewed characteristics, ranging from 0 to 14.70, with one peak lying at the score of two (Fig. 1A). Females had a comparable burden of multimorbidity as males (Fig. 1B). Those aged 70 + turned to have a relatively higher burden of multimorbidity (Fig. 1C).

**Fig. 2:**
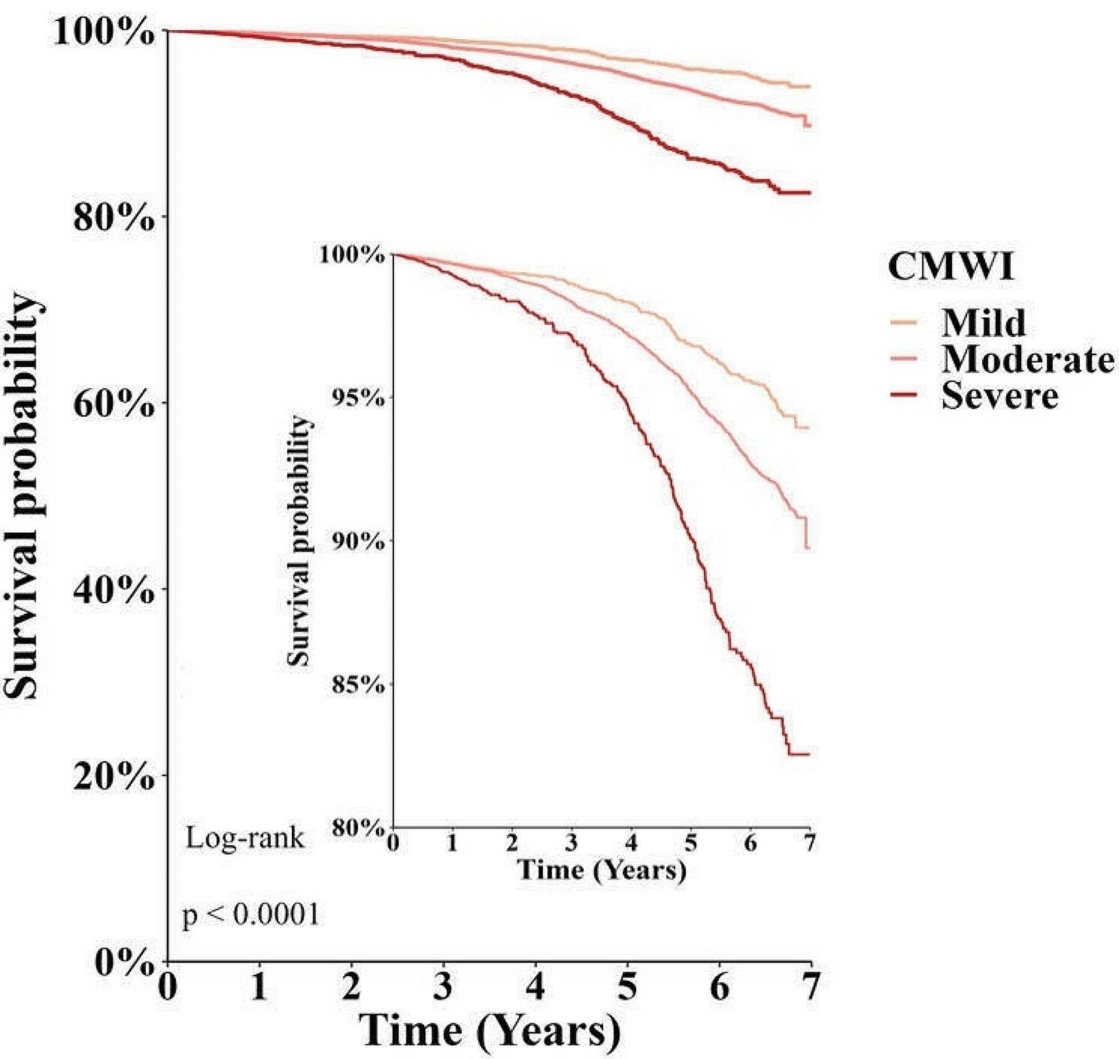
Seven-year Kaplan-Meier survival curve for mortality for categories of CMWI

The Cox regression results of mortality were shown in Table 2. CMWI was associated with increased mortality, such that one-point increase in baseline CMWI increased the mortality risk by 18% (*HR* = 1.18, 95% *CI* = 1.14, 1.22, *P* < 0.001). A dose-response relationship existed between mortality hazard ratios and CMWI categories. Compared to the bottom tertile, individuals in the middle tertile and top tertile of CMWI had 132% (*HR* = 1.32, 95%*CI* = 1.12, 1.55, *P* < 0.001) and 252% (*HR* = 2.52, 95% *CI* = 2.07, 3.05, *P* < 0.001) higher mortality risks, respectively (Table 2). Kaplan–Meier curves of all-cause mortality stratified by CMWI categories also demonstrated a significant higher all-cause mortality with higher CMWI levels (Log-rank *P* < 0.0001, Fig. 2). In a sensitivity analysis of the relationship between CMWI (continuous and categorical) and mortality, the finding was similar to the main analysis for mortality (Supplementary Table 4). The CMWI was associated with increased mortality, such that one-point increase in baseline CMWI increased the morality risk by 17% (*HR* = 1.17, 95% *CI* = 1.13, 1.21, *P* < 0.001). The results of our sensitivity analyses within the older age group, which were consistent with the main model analysis, suggest the robustness of our main findings.

The logistic regression results of disability were shown in Table 2. CMWI was associated with increased disability, such that one-point increase in baseline CMWI increased the disability risk by 12% (*OR* = 1.12, 95% *CI* = 1.05, 1.20, *P* < 0.01). The similar dose-response relationship was observed between disability odds ratios and CMWI categories. The odds of disability were estimated to be 90% higher in the severe category compared to the mild category (*OR* = 1.99, 95% *CI* = 1.16, 3.63, *P* < 0.05), although statistical significance was not attained in the moderate category (*OR* = 1.56, 95% *CI* = 0.94, 2.79, *P* = 0.11).

In addition, we compared the validity of CMWI and other multimorbidity indices for predicting the two health outcomes. For mortality, CMWI showed a better validity with a lowest AIC (21,342) and same C-statistic (0.76). For disability, CMWI demonstrated the better model fit with smaller AIC and comparable prediction performance, as both the continuous and categorical forms had the comparable AUC (0.84), and the smaller AIC (2,186 and 2,191, respectively) (Supplementary Tables 5& 6).

## Discussion

We assessed the validity of CMWI in quantifying disease burden and associated of disability and mortality for Chinese older adults, utilizing routinely collected health check-ups data of primary care. We found half of these older adults investigated had multimorbidity burden below 1.70 as measured by CMWI, with one unit increase in CMWI associated with 12% and 18% increased risks in subsequent disability and mortality, respectively. Adequate model fits support the application of CMWI to primary care data.

Our study contributes to evidence on multimorbidity burden for Chinese older adults in primary care settings. The weights of chronic diseases in the CMWI are developed on cohort data representative of middle-aged and older adults in Chinese communities [24], whereas other indices such as ECI [16] and CCI [17] are based on western inpatient data. Studies have shown that the CMWI is an adequate index tailored to Chinese middle-aged and older community-dwelling individuals [38, 39]. Furthermore, compared to indices like ECI and CCI, the CMWI demonstrated better model fit and comparable predictive performance in this study (Supplementary Tables 5& 6), thus validating this easily computed measure of multimorbidity at primary care setting. Compared with previous studies that used prevalence of multimorbidity (i.e., ≥ 2 chronic diseases) [12] or simply count [13] to measure multimorbidity burden in primary care settings, our study provides a more sensitive measure to quantify disease burden by applying CMWI with chronic conditions weighted by physical functioning. Prior evidence has demonstrated that diverse chronic conditions have varied effects on physical functioning which is strongly associated with increased mortality risk [40]. Our weight of CMWI indicates relative greater effects of stroke and dementia to health and quality of life among 14 chronic diseases on Chinese middle-aged and older adults [24]. In most previous studies on multimorbidity burden in primary care [8, 41], multimorbidity was typically defined qualitatively as the presence of two or more chronic diseases. In contrast, this study uses the CMWI for quantitative measurement, applying it to primary health check-up data to describe the multimorbidity burden and predict health outcomes, thereby providing an evidence base for a most appropriate multimorbidity measure in primary care scenarios. Our findings indicated that the CMWI has a broader distribution than previous using count as a measurement in capturing individual multimorbidity in primary care [12, 13], which allows for finer differentiation among disease burden of individuals and thus provides a more sensitive measure suitable for Chinese aging population. The CMWI provides valid measurements of the varying degrees of disease burden among patients with the same count of chronic diseases but differing types, which were not addressed by the simple disease count. Applying CMWI to measure disease burden can be more informative for individuals and better describe the distribution of multimorbidity in primary care settings.

Our study revealed that the CMWI is a valid measurement to monitor disease burden and to detect associated health risks for older adults in primary care settings. The three key advantages of applying CMWI to measure multimorbidity burden in health check-ups data at primary care setting for Chinese older adults are providing early warnings, risk stratification and easy to use. First, for adverse outcomes of multimorbidity, our study confirms the multimorbidity and increased mortality risk and extends prior studies results by including disability [42, 43]. The CMWI showed better model fits with smaller AIC and comparable prediction performances for both mortality and disability risks in primary care settings (Supplementary Tables 5& 6), compared with the other four multimorbidity indices [24]. This finding could lie in the weights of CMWI tailored for Chinese based on impacts of chronic diseases on physical functioning which is a relatively early warning outcome than mortality for older adults. Second, in addition to continuous CMWI, the categorical CMWI might be more valuable for risk stratification. Individuals in the top tertile of CMWI had the highest disability and mortality risks. This suggests the potential use of the CMWI as an individual-level risk stratification tool in routine health check-ups in primary care settings. Finally, the CMWI can be easily calculated by disease inventories in electronic health records without additional data collection for primary care providers. The annual health check-up is the most basic service provided free by the national basic public health service programmes in China [10]. Moreover, the coverage of electronic health records had been over 85% in 2016 in China [11]. Besides, studies have demonstrated that individuals with multimorbidity face increased complexity in healthcare services [44, 45], which may undermine the coordination, continuity, and safety of medical care. Instead of a one-size-fits-all approach, a patient-centred multimorbidity measurement that differentiates levels of multimorbidity and understands patient-specific needs and priorities within the primary care is likely more beneficial [46]. Integrating the identification and assessment of multimorbidity into routine management in primary care settings may help in risk-stratifying older adults with a relatively high CMWI score for follow-up or appropriate medical services. Taken together, the CMWI could be easy to use, provide early warnings and facilitate targeted health management plans for older adults at high-risks in routine health check-ups in primary care settings.

The main strength of our study is its use of the CMWI, developed using the ageing cohorts representative of Chinese middle-aged and older community-dwelling individuals, to measure multimorbidity burden in annual health check-ups data of primary care. There are also several limitations. First, our study was conducted only in regional routine health check-ups data. Nevertheless, the CMWI that developed and validated using ageing cohorts representative of the Chinese population. The health check-up system in the study region is relatively complete and informative. Similarly, regions with well-established electronic health check-up record systems can similarly benefit from this application. Conversely, in areas where these systems are underdeveloped, application may be less feasible. However, as national electronic health check-up records are gradually being improved [11], expanding validation to a broader range of nationwide primary care settings would be useful. Besides, we recognised that our study outcomes were focused on disability and mortality, which should be broadened in future studies to reflect a wider array of relevant outcomes for older individuals such as quality of life, healthcare utilisation and costs. Furthermore, we only focused on measuring the burden of multimorbidity at single time points, rather than considering the longitudinal and additive nature of chronic conditions, even though the annual health check-up data provide frequent individual-level longitudinal health records. The dynamic monitor of multimorbidity burden in real world should be considered [18]. Finally, while lifestyle behaviors like smoking and alcohol consumption are known risk factors for chronic diseases [47, 48], we didn’t include them in the modeling analysis. The aim of this study is to evaluate the validity of the CMWI in measuring individual multimorbidity burden for risk stratification, utilizing annual health check-up data at the primary care level, and adjusting for basic factors such as age and sex. Given that more than half of the records for smoking and alcohol consumption among older adults were missing, adjusting for these risk factors is impractical for the current data. With improvements in annual health check-up records, future research can incorporate these factors.

## Conclusion

We found that applying CMWI to routine health check-ups could quantify the multimorbidity burden and detect disability and mortality risks. It is a valid tool for primary care providers to quantify and monitor health risks and to facilitate targeted health management plans for older patients in primary care.

### Electronic supplementary material

Below is the link to the electronic supplementary material.

Supplementary Material 1

## Data Availability

The datasets used and/or analysed during the current study are available from the corresponding author on reasonable request.

## Abbreviations

ECI: Elixhauser Comorbidity Index
CCI: Charlson Comorbidity Index
MWI: Multimorbidity-Weighted Index
CMWI: Chinese Multimorbidity-Weighted Index
CHARLS: China Health and Retirement Longitudinal Study
IQR: Inter-Quartile Range
OR: Odds Ratio
HR: Hazard Ratio
CI: Confidence Interval
AIC: Akaike Information Criterion
AUC: Area Under the receiver operating characteristic Curve
