# Supplementary material for: Validity of the Chinese multimorbidity-weighted index in measuring disease burden using health check-ups data in primary care
Source: BMC Public Health. 2024 Jul 26;24:1999. doi: 10.1186/s12889-024-19479-6 (PMC11282735; doi:10.1186/s12889-024-19479-6)
Supplement: Supplementary file 1 — Supplementary Material 1 [file 12889_2024_19479_MOESM1_ESM.docx]

**Supplementary material**

**Title:**

**Validity of the Chinese Multimorbidity-Weighted Index in Measuring Disease Burden Using Health Check-ups Data in Primary Care**

**Contents:**

**Supplementary Figure 1.** The flow chart of the selection of study population

**Supplementary Table 1**. Differences between the populations included in the disability analysis and those with missing values

**Supplementary Table 2.** Chinese multimorbidity-weighted index weightings for 14 chronic diseases and conditions

**Supplementary Table 3.** Interaction analysis for age and CMWI

**Supplementary Table 4.** The performance of CMWI for predicting mortality in 70+ older adults

**Supplementary Table 5.** The performance of different multimorbidity indexes for predicting mortality in 65+ older adults (N=45,009)

**Supplementary Table 6.** The performance of different multimorbidity indexes for predicting disability in 65+ older adults (N=18,230)

**Supplementary Figure 1.** The flow chart of the selection of study population


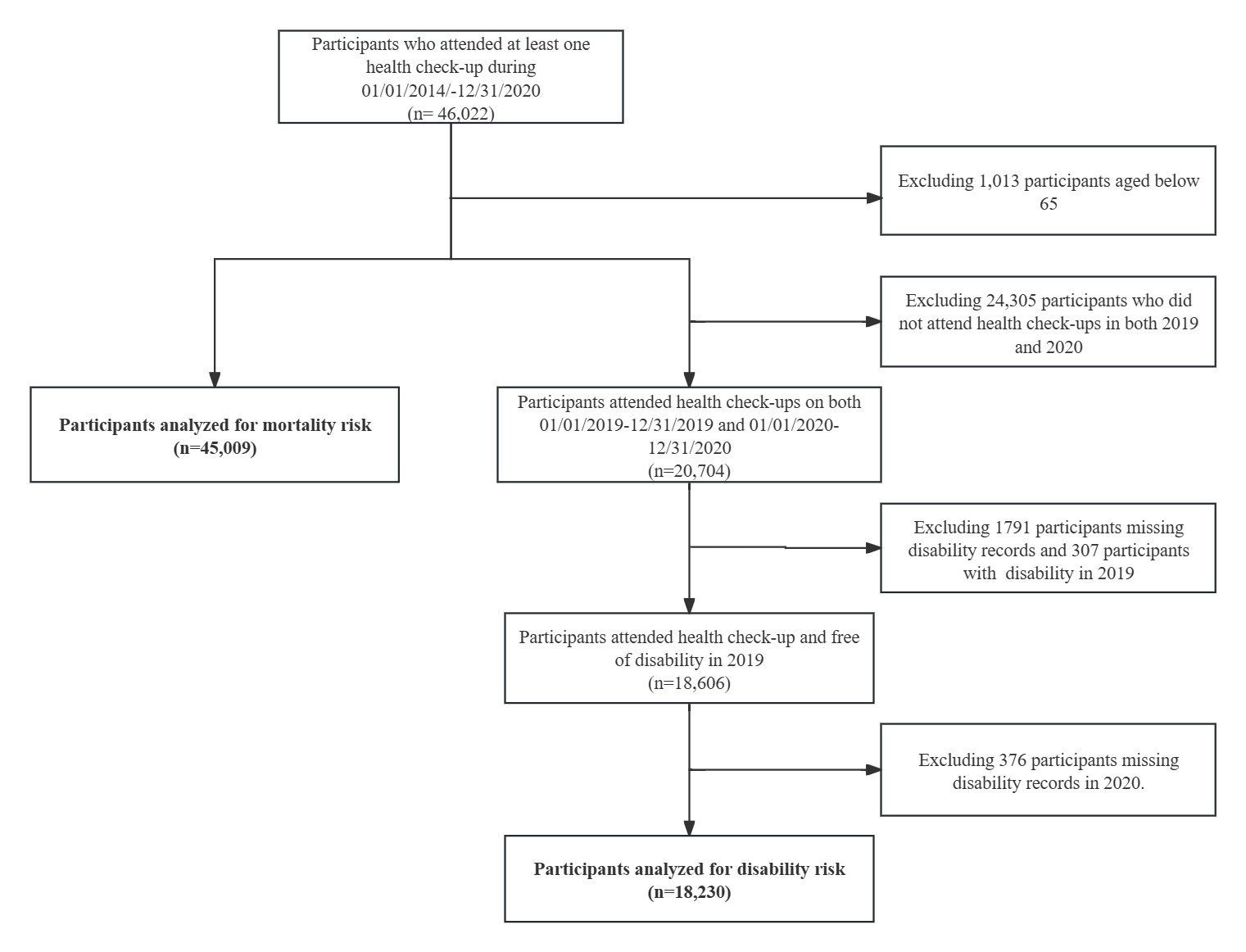


Supplementary Figure 1. The flow chart of the selection of study population.

**Supplementary Table 1**. Differences between the populations included in the disability analysis and those with missing values

| Variable | Statistic (*W* or ^2^) | *P* value |
| --- | --- | --- |
| Age | 170275988 | 0.503 |
| Sex | 0.18318 | 0.669 |
| CMWI | 170292075 | 0.493 |

**Supplementary Table 2.** Chinese multimorbidity-weighted index weightings for 14 chronic diseases and conditions

| Chronic conditions | Weights of CMWI |
| --- | --- |
| Stroke | 5.1 |
| Memory-related disease (e.g., dementia, brain atrophy, Parkinson’s disease) | 4.3 |
| Cancer or malignant tumour (excluding minor skin cancers) | 3.4 |
| Asthma | 2.4 |
| Arthritis or rheumatism | 2.2 |
| Emotional, nervous, or psychiatric problems | 2.1 |
| Heart disease (e.g., coronary heart disease, angina, congestive heart failure) | 1.7 |
| Chronic lung diseases (e.g., chronic bronchitis, emphysema, excluding tumours or cancer) | 1.6 |
| Hypertension | 1.3 |
| Kidney disease (except for tumour or cancer) | 1.1 |
| Diabetes or high blood sugar | 1.0 |
| Stomach or other digestive disease (except for tumour or cancer) | 0.7 |
| Dyslipidaemia (e.g., elevation of total cholesterol) | 0.2 |
| Liver disease (except fatty liver, tumours, and cancer) | 0.2 |

| **Multimorbidity index** | ***HR* (95%CI)** | ***P*** | ***C*-statistic** |
| --- | --- | --- | --- |
| CMWI, continuous | 1.17 (1.13,1.21) | <0.001 | 0.74 |
| CMWI, categories |  |  |  |
| Severe (≥3.80) | 2.45 (1.98,3.04) | <0.001 | 0.74 |
| Moderate (≥1.30) | 1.28 (1.07,1.54) | <0.001 |  |
| Mild (reference) | - | - |  |

**Supplementary Table 3** Interaction analysis for age and CMWI

**Supplements Table 4** The performance of CMWI for predicting mortality in 70+ older adults

| outcomes | Interaction terms | *P* value |
| --- | --- | --- |
| Mortality | Age× CMWI | 0.001 |
| Disability | Age× CMWI | 0.719 |

**Supplements Table 5** The performance of different multimorbidity indexes for predicting mortality in 65+ older adults (N=45,009)

| **Multimorbidity index** | ***HR* (95%CI)** | ***P*** | **AIC** | ***C*-statistic** |
| --- | --- | --- | --- | --- |
| CMWI, continuous | 1.18 (1.14,1.22) | <0.001 | 21,342 | 0.76 |
| CMWI, categories |  |  |  |  |
| Severe (≥3.80) | 2.52 (2.07,3.05) | <0.001 | 21,342 | 0.76 |
| Moderate (≥1.30) | 1.32 (1.12,1.55) | <0.001 |  |  |
| Mild (reference) | - | - |  |  |
| MWI, continuous | 1.13 (1.10,1.15) | <0.001 | 21,353 | 0.76 |
| MWI, categories |  |  |  |  |
| Severe (≥5.41) | 2.60 (2.10,3.21) | <0.001 | 21,355 | 0.76 |
| Moderate (≥1.53) | 1.47 (1.23,1.75) | <0.001 |  |  |
| Mild (reference) |  |  |  |  |
| ECI, continuous | 1.29 (1.22,1.36) | <0.001 | 21,357 | 0.76 |
| ECI, categories |  |  |  |  |
| Severe (≥2) | 1.80 (1.50,2.16) | <0.001 | 21,388 | 0.76 |
| Moderate (≥1) | 1.38 (1.14,1.67) | <0.001 |  |  |
| Mild (reference) | - | - | - |  |
| CCI, continuous | 1.20 (1.15,1.24) | <0.001 | 21,354 | 0.76 |
| CCI, categories |  |  |  |  |
| Severe (≥2) | 1.81 (1.51,2.18) | <0.001 | 21,384 | 0.76 |
| Moderate (≥1) | 1.36 (1.12,1.65) | <0.01 |  |  |
| Mild (reference) | - | - | - |  |
| Count, continuous | 1.23 (1.17,1.29) | <0.001 | 21,366 | 0.76 |
| Count, categories |  |  |  |  |
| Severe (≥3) | 2.06 (1.66,2.55) | <0.001 | 21,383 | 0.76 |
| Moderate (≥1) | 1.41 (1.16,1.72) | <0.001 |  |  |
| Mild (reference) | - | - | - |  |

*Abbreviations*: CMWI, Chinese multimorbidity-weighted index; MWI, multimorbidity-weighted index; ECI, Elixhauser comorbidity index; CCI, Charlson comorbidity index; HR, hazard ratio; CI, confidence interval; AIC, Akaike information criterion; C, concordance

**Supplements Table 6** The performance of different multimorbidity indexes for predicting disability in 65+ older adults (N=18,230)

| **Multimorbidity index** | ***OR* (95%CI)** | ***P*** | **AIC** | | **AUC** |
| --- | --- | --- | --- | --- | --- |
| CMWI, continuous | 1.12 (1.05,1.20) | <0.01 | | 2,186 | 0.84 |
| CMWI, categories |  |  | |  |  |
| Severe (≥3.80) | 1.99 (1.16,3.63) | <0.05 | | 2,191 | 0.84 |
| Moderate (≥1.30) | 1.56 (0.94,2.79) | 0.11 | |  |  |
| Mild (reference) | - | - | |  |  |
| MWI, continuous | 1.07 (1.02,1.12) | <0.01 | 2,189 | | 0.83 |
| MWI, categories |  |  |  | |  |
| Severe (≥5.41) | 1.91 (1.11,3.50) | <0.05 | 2,192 | | 0.83 |
| Moderate (≥1.53) | 1.49 (0.90,2.67) | 0.15 |  | |  |
| Mild (reference) |  |  |  | |  |
| ECI, continuous | 1.12 (1.01,1.25) | <0.05 | 2,191 | | 0.83 |
| ECI, categories |  |  |  | |  |
| Severe (≥2) | 1.52 (0.87,2.92) | 0.17 | 2,193 | | 0.83 |
| Moderate (≥1) | 1.17 (0.64,2.30) | 0.64 |  | |  |
| Mild (reference) |  |  |  | |  |
| CCI, continuous | 1.09 (1.01,1.17) | <0.05 | 2,191 | | 0.83 |
| CCI, categories |  |  |  | |  |
| Severe (≥2) | 1.48 (0.85,2.83) | 0.20 | 2,195 | | 0.83 |
| Moderate (≥1) | 1.22 (0.67,2.41) | 0.55 |  | |  |
| Mild (reference) |  |  |  | |  |
| Count, continuous | 1.04 (0.95,1.14) | 0.41 | 2,195 | | 0.83 |
| Count, categories |  |  |  | |  |
| Severe (≥3) | 1.63 (0.72,4.67) | 0.30 | 2,196 | | 0.83 |
| Moderate (≥1) | 1.52 (0.67,4.37) | 0.37 |  | |  |
| Mild (reference) |  |  |  | |  |

*Abbreviations*: CMWI, Chinese multimorbidity-weighted index; MWI, multimorbidity-weighted index; ECI, Elixhauser comorbidity index; CCI, Charlson comorbidity index; OR, odds ratio; CI, confidence interval; AIC, Akaike information criterion; AUC, the area under the receiver operating characteristic curve
